# Relationship between Product and Process Characteristics of Handwriting Skills of Children in the Second Grade of Elementary School

**DOI:** 10.3390/children10030445

**Published:** 2023-02-24

**Authors:** Helena Coradinho, Filipe Melo, Gabriela Almeida, Guida Veiga, José Marmeleira, Hans-Leo Teulings, Ana Rita Matias

**Affiliations:** 1 Comprehensive Health Research Centre (CHRC), Department of Sport and Health, School of Health and Human Development, University of Évora, 7004-516 Évora, Portugal; 2 Comprehensive Health Research Centre (CHRC), Faculty of Human Kinetics, University of Lisbon, 1649-004 Lisboa, Portugal; 3 NeuroScript, Tempe, AZ 85282, USA

**Keywords:** handwriting quality and speed, product and process, school-age children, kinematic analysis of handwriting, dysgraphia

## Abstract

The purpose of this study was to analyze the relationship between the quality and speed of handwriting and the process characteristics of the handwriting of children in the second grade of elementary school considered as a function of age and gender. A sample of 57 children (mean age 7.25 years, SD 0.43) participated in the study. The Concise Assessment Method for Children’s Handwriting (BHK) was used to assess the quality and speed of handwriting. The characteristics of the process of handwriting were assessed using MovAlyzeR^®^ software. The handwriting of boys showed a significantly greater number of strokes and slanted more to the right than the handwriting of girls. Handwriting quality and speed significantly correlated with several process characteristics: the number of strokes, reaction time, duration, relative pen-down duration, average pen pressure, vertical size, horizontal size, road length, and average absolute velocity. This research contributes to the construction of normative values in the process characteristics of the handwriting of elementary school children and provides a promising step towards the early identification of difficulties that can lead to dysgraphia, thus preventing later difficulties in handwriting.

## 1. Introduction

Handwriting skills are crucial for academic success [1,2]. During the school day, children spend, on average, 33% of their time engaged in fine motor activities, of which 85% is handwriting [3]. However, a significant proportion (12 to 30%) of children have handwriting difficulties [4]. Furthermore, handwriting difficulties are among the most common reasons that school children attend psychomotor therapy [5]. For this reason, it is important to understand handwriting development in typically developing children, in terms of both product and process, in order to identify the early signs of handwriting difficulties in an attempt to remediate them [2,6,7,8,9,10]. Remediation is important for both the present and the future, as it can allow for the possibility of more precisely and efficiently diagnosing dysgraphia. Furthermore, identifying the main characteristics that impact the quality and speed of the handwriting process is essential for developing more effective interventions [8].

Handwriting is a complex skill that children develop over several years [7,11,12]. Formal handwriting instruction is typically introduced in the first grade (ages 6–7) [13]. In that grade, the quality of the handwriting improves significantly and then reaches a plateau in the second grade (ages 7–8) [7,12,14]. In the third grade (ages 8–9), the quality of the handwriting improves further, as handwriting becomes more automatic. Overall, handwriting speed and quality progressively increase during elementary school [7,12,14,15,16].

Several studies have examined the relationship between handwriting quality and speed, but they show divergent results [17]. Although some studies suggest no relationship between the quality and speed of handwriting [18,19,20,21], others suggest some relationship [18,22,23]. For example, Weintraub and Graham showed that, when children were specifically asked to handwrite neatly, the speed of their handwriting decreased. Inversely, when the children were specifically asked to handwrite quickly, the legibility of their handwriting decreased [23].

Likewise, studies show conflicting results with respect to handwriting as a function of gender [13]. Some studies show that girls’ handwriting is better and faster than that of boys [14,15,16,24,25], while others find gender differences in the quality and speed of handwriting [19,26,27].

Traditional studies of handwriting performance have been restricted to evaluating the product, i.e., the mastering of handwriting quality (or legibility) and speed (in terms of the number of characters produced per minute), using pencil-and-paper tests [28,29,30,31]. In recent years, the assessment of handwriting skills has significantly evolved. Currently, the evaluation of the product of handwriting performance is complemented by an evaluation of the process of handwriting: measuring pressure and speed by collecting spatial, temporal, and kinematic data on handwriting performance [28,30,31,32,33]. This process evaluation is obtained in real-time, as the movements are produced with the aid of a digitizing tablet, a pen, and specific software. Therefore, in this study, the evaluation of the product of handwriting performance using the Concise Assessment Method for Children’s Handwriting ((Beknopte Beoordelingsmethode voor Kinderhandschriften), BHK) [34] is complemented by evaluating the process of handwriting while copying the BHK text of increasingly complex words for 5 min. Studying the characteristics of the process of handwriting performance is essential because there are a lack of normative data on the typical development of the skills involved [9,35]. Collecting this type of data is also important because these data serve as a reference for the early identification of handwriting difficulties, and they help in assessments by allowing for more objective diagnoses of handwriting difficulties and the development of more effective interventions [35].

Previous studies in typically developing children from the first and second grades of primary schools have shown that the duration of handwriting strokes [6,9,35,36,37], the number of strokes [6,38], the duration of pauses [38,39], the vertical and horizontal sizes, and the slant of the handwriting [37] tend to decrease with age, while pen velocity tends to increase with age [9,37,39,40,41]. However, there is no consensus on handwriting pressure [9,35] or road length [6,9]. Some studies report a decrease in handwriting pressure [37,41] and road length [6] as age increases, while others report the absence of any relationship between handwriting pressure [9,39] and road length [9] as a function of age.

The studies on gender differences in graphomotor process characteristics are even more limited, and they do not arrive at any consensus. Some studies did not find gender differences in handwriting pressure [36,42], duration [36], or velocity [42], while others report differences in favor of females in duration, road length, and velocity [43].

The relationship between the characteristics of the graphomotor product and the graphomotor process needs to be studied further. A study by Gargot et al. [8] indicates a significant association between airtime (the duration of a pause where the pen is lifted) and handwriting quality and speed (the number of characters written in 5 min) as measured using the BHK text. In this case, a shorter airtime was associated with a higher quality and speed of handwriting in children from the first to fifth grades [8]. However, no correlations were found between the quality and speed of the handwriting or handwriting pressure or slant [8]. Vaivre-Douret et al. [37] examined drawing tasks in children from the first to fifth grades and found a significant correlation between the quality and speed of handwriting as expressed by the number of characters produced in 5 min according to the BHK and the vertical size, horizontal size, slant, duration, and average velocity of the handwriting strokes. Although we live in an age of technology, where handwriting has been dropped from the mandatory part of school curricula in several countries (e.g., the USA and Finland) and replaced by typing, this trend has begun to reverse, as several studies have shown the importance of automated handwriting skills for achieving the highest academic goals [44,45]. Therefore, now more than ever, it is vital to study the characteristics of the handwriting process and its relationships with the product of the process. Through this approach and after collecting a larger corpus of data, we may be able to extract product and process characteristics from handwriting and train artificial neuron networks (ANNs) to effectively identify children who are likely, or who are unlikely, to develop dysgraphia [46].

## 2. Materials and Methods

### 2.1. Ethics Approval

The study proposal was authorized by the Directorate General for Education. The present study was approved by the university’s Ethics Committee for Research in the Areas of Human Health and Welfare. Informed consent was obtained from the parents, and verbal assent was given by the children.

### 2.2. Participants

A total of 57 children (22 boys and 35 girls; mean age 7.25, SD 0.43) from public schools in the district of Évora (Portugal) participated in this study. Many parents (i.e., 73 or 56%) did not consent to the participation of their children in this study, so their children were not included.

Most of the children (i.e., 47 or 82.5%) were right-handed, 6 (10.5%) were left-handed, and 4 (7%) were ambidextrous. With regard to pen-hold, 30 children (52.6%) showed a tridigital grip, 22 (38.6%) a tripod grip, and 5 (8.8%) a lateral grip. With regard to the socioeconomic level of the family, 28 were from upper-middle-class (49.1%), 17 were from middle-class (29.8%), and 12 were from upper-class (21.5%) families.

The inclusion criteria specified children with typical development in the second grade of elementary school. The exclusion criteria were as follows: a diagnosis or suspicion of neurological disabilities (e.g., cerebral palsy, epilepsy); psychiatric and/or behavioral disorders; the presence of uncorrected vision and hearing problems; referral by the Special Education Service; the presence of one or more school retentions; Portuguese not being the mother tongue; and previous participation in a similar study.

The data of 20 children were excluded from the analysis based on these exclusion criteria. They were evenly spread across the groups. In addition, technical problems with MovAlyzeR^®^ occurred with 3 children, who were therefore excluded from the analysis.

### 2.3. Instruments

#### 2.3.1. Concise Assessment Method for Children’s Handwriting (BHK)

The BHK (Portuguese version) was used to measure product characteristics, specifically the quality and speed of handwriting. The BHK test involves 5 min of copying a text. The higher the total score, the lower the handwriting quality. A total score below 21 indicates that the child has no handwriting difficulties; a total score between 22 and 28 corresponds with poor performance, and a total score of more than 29 reveals very poor handwriting [15]. The internal consistency of the Portuguese version shows an acceptable Cronbach’s coefficient alpha value of 0.64 [47].

#### 2.3.2. Equipment

To assess process characteristics, handwriting movements were recorded using an x-y digitizing tablet (WACOM, Intuos Pro L, model PTH-851) with an inking pen (WACOM Inking pen, model KP-130-01) and MovAlyzeR^®^ software (version 6.1, NeuroScript, LLC; Tempe, AZ, USA) run on an MS Windows laptop computer connected to the tablet via USB. The tablet had an active surface area of 32.51 cm × 20.32 cm, a device resolution of 0.0005 cm, and a sampling frequency of 100 Hz. The tablet was covered with an A4 sheet of paper (29.7 cm × 21 cm) in vertical orientation and taped onto the tablet.

**Figure 1:**
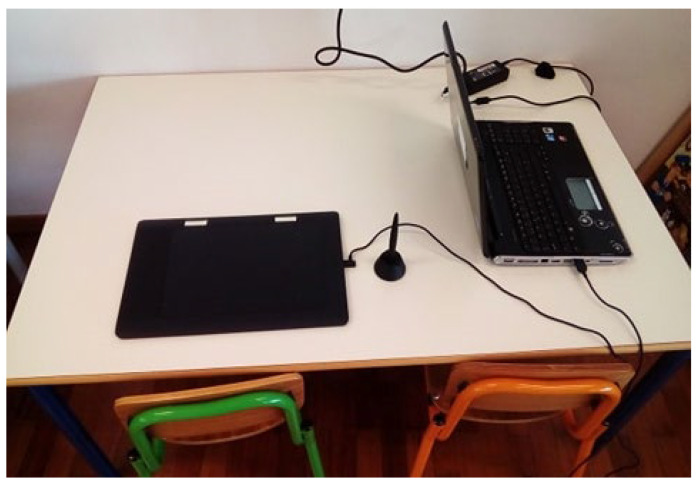
The experimental graphomotor setup with a pen tablet, school desk, and chairs. The experimenter was seated on the right side and could monitor the pen movements in real time on a laptop.

The participants were seated in front of the tablet, which was centered on the participant’s midline in front of their chest. The heights of the chair and table were adjusted, and the laptop computer was set aside so that the real-time visual feedback of the child’s pen movements was available only to the experimenter (see Figure 1). The participants were invited to copy the BHK text for 5 min.

The following handwriting measures were extracted [48]:

(a) Temporal measures:
  – *Duration* is the time interval in seconds between the beginning of the first stroke and the end of the last stroke.
  – *Start Time* is the reaction time in seconds measured as the time interval between the stimulus onset and the start of the first stroke.
  – *Relative Pen-Down Duration* is the proportion of the duration that the pen is on the paper relative to the total duration per handwriting stroke.
  – *Number of Strokes* is the number of upward or downward strokes that a child performs while using the BHK copy.
(b) Spatial measures:
  – *Vertical Size* is the vertical distance in centimeters between the beginning and end of a stroke.
  – *Horizontal size* is the horizontal distance in centimeters between the beginning and end of a stroke.
  – *Road Length* or path length is the length in centimeters of a stroke trajectory from beginning to end.
  – *Slant* is the direction in radians from the beginning point to the endpoint of a stroke (horizontal to rightward is 0 radians; 60 degrees to the right is 1.05 radians; 90 degrees (therefore upright) is 1.57 radians).
(c) Kinematic measures:
  – *Average Absolute Velocity* is the mean speed of movement in centimeters per second across all stroke samples.
(d) Kinetic measures:
  – *Average Pen Pressure* is the mean axial force exerted on the pen across a stroke in pen tablet units (a normal pen pressure of 400 corresponds to 100 g of force).

### 2.4. Procedure

**Figure 2:**
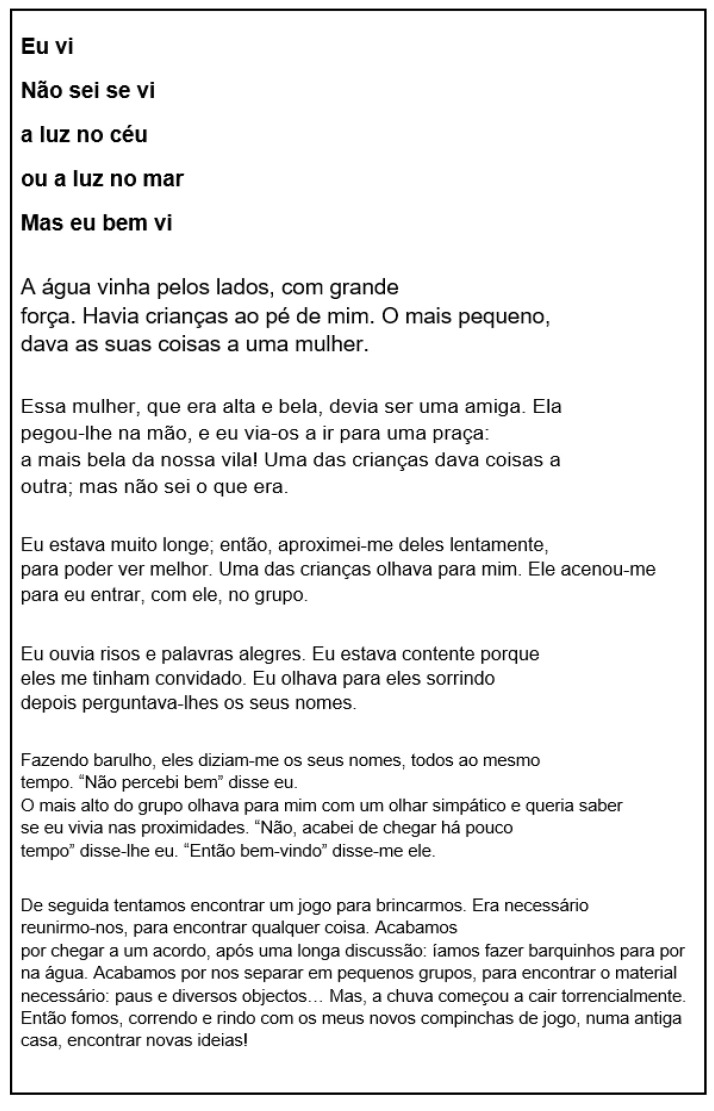
BHK text (Portuguese version obtained from the French version [15]—Figure A1 in Appendix A). Participants copied the BHK text, composed of progressively more complex words, for 5 min.

**Figure 3:**
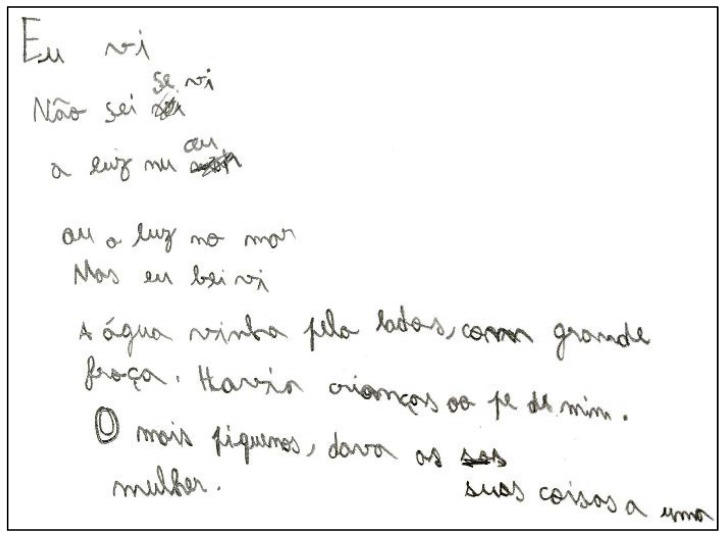
Handwriting example of the BHK text (Portuguese version is shown in Figure 2 and the English translation is shown in Appendix A) copied for 5 min by a participant on a blank A4 sheet of paper in landscape orientation. (Image was reduced and cropped. The border measured in reality 18.4 cm × 13.5 cm.)

We recruited children from seven elementary schools in the district of Évora. All participants were individually tested in a quiet room in the school. The testing was carried out by the assistant principal investigator, who is a trained examiner. The participant was seated on a standard school chair at a school desk, which was appropriate for their height. The BHK text (see Figure 2) was copied for 5 min using an electronic inking pen on a sheet of paper positioned on the digitizing table (see Figure 3). Pen grip and hand posture were photographed in three different directions (front, right, and left). Data collection took about 10 min per child.

### 2.5. Statistical Analysis

Data were analyzed using the Statistical Package for the Social Sciences, version 19.0, for Windows (SPSS Inc., Chicago, IL, USA). Tests of normality and descriptive statistics were performed for all outcome variables as a function of age and gender. Differences in the mean values between the groups for all normally distributed measures were examined using an independent-samples *t*-test. The measures that did not meet the normal distribution assumptions were compared using the nonparametric Mann–Whitney U test. Statistical significance was set at *p* ≤ 0.05. Lastly, to analyze the relationship among all variables, Spearman’s correlation coefficients were calculated. The strength of the relationship was set at r < 0.25 indicating a weak effect, r = 0.25–0.5 a moderate effect, r = 0.5–0.75 a strong effect, and r > 0.75 a very strong effect [49].

## 3. Results

### 3.1. Group Differences

#### 3.1.1. Handwriting Product Characteristics

For the BHK, no significant differences in handwriting quality and speed were found between the age groups (Table 1).

#### 3.1.2. Handwriting Process Characteristics

The results indicate no significant differences between the age groups in the process characteristics of handwriting (Table 1).

### 3.2. Gender Differences

#### 3.2.1. Handwriting Product Characteristics

No gender differences were found in handwriting quality and speed (Table 1).

#### 3.2.2. Handwriting Process Characteristics

**Figure 4:**
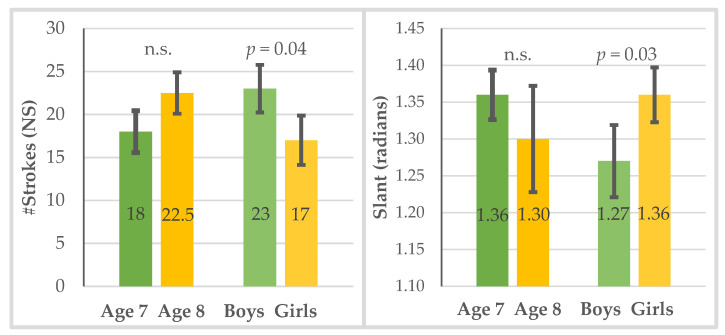
The only significant group differences are between boys and girls for the number of strokes (left panel) and the slant in radians (right panel) (1.27 rad = 73 deg, 1.36 rad = 78 deg).

The boys (Mdn = 23.00) presented a significantly higher number of strokes than the girls (Mdn = 17.00) (U = 260.50, *p* < 0.05) (see Table 1 and Figure 4, left panel), and the girls (Mdn = 1.36) presented a significantly higher slant than the boys (Mdn = 1.27) (U = 254.00, *p* = 0.03); i.e., the girls wrote more upright, while the boys wrote more slanted to the right (see Table 1 and Figure 4, right panel).

Finally, no gender differences were found for the other features (i.e., reaction time, duration, relative pen-down duration, average pen pressure, vertical size, horizontal size, road length, and average absolute velocity).

### 3.3. Relationship between Age and Product and Process Handwriting Characteristics

#### 3.3.1. Correlation between Handwriting Quality and Speed

No significant association was found between handwriting quality and speed for the 7-year-old group (r = −0.14, *p* = 0.38), the 8-year-old group (r = −0.10, *p* = 0.75), or the total group (r = −0.19, *p* = 0.17) (see Table 2).

#### 3.3.2. Correlation between the Handwriting Produced and the Process Characteristics

The correlation analysis results are shown in Table 2. In the total population, we found positive correlations between handwriting quality and reaction time (r = 0.38, *p* < 0.05), duration (r = 0.32, *p* < 0.05), vertical size (r = 0.50, *p* < 0.01), horizontal size (r = 0.43, *p* < 0.01), path length (r = 0.50, *p* < 0.01), and average absolute velocity (r = 0.49, *p* < 0.01). Thus, in the population of 7- and 8-year-olds, a high handwriting quality score was correlated with a greater reaction time, longer duration, larger vertical size, larger horizontal size, larger path length, and greater average absolute velocity.

While the 8-year-olds did not show any significant correlations between handwriting quality and any of the process variables, we found several positive correlations in the 7-year-olds: between handwriting quality and the number of strokes (r = 0.35, *p* < 0.05) and between handwriting quality and duration (r = 0.45, *p* < 0.05). Thus, in the 7-year-olds, the higher the handwriting quality score, the higher the number of strokes and the longer the duration per stroke. A strong positive correlation was also found between handwriting quality and reaction time (r = 0.53, *p* < 0.01), vertical size (r = 0.63, *p* < 0.01), horizontal size (r = 0.61, *p* < 0.01), path length (r = 0.64, *p* < 0.01), and average absolute velocity (r = 0.56, *p* < 0.01). Thus, in the 7-year-olds, a high handwriting quality was associated with a longer reaction time, larger vertical size, larger horizontal size, and larger average absolute velocity. We also found negative correlations between handwriting quality and relative pen-down duration (r = −0.40, *p* < 0.05) and between handwriting quality and average pen pressure (r = −0.38, *p* < 0.05). Thus, a high handwriting quality was associated with a low relative pen-down duration and a low average pen pressure.

Unlike BHK handwriting quality, the speed of handwriting showed no significant correlations.

### 3.4. Relationship between Variables according to Gender

#### 3.4.1. Correlations between Handwriting Quality and Speed

No significant correlation was found between handwriting quality and speed for the boys (r = −0.31, *p* = 0.16) or for the girls (r = −0.12, *p* = 0.49) (Table 3).

#### 3.4.2. Correlations between the BHK and the Process Characteristics of the Handwriting

The results for the boys indicate a significant strong positive correlation between handwriting quality and vertical size (r = 0.52, *p* < 0.05) and between handwriting quality and average absolute velocity (r = 0.57, *p* < 0.05) (Table 3). Thus, high handwriting quality scores were associated with a larger vertical size and a greater average absolute velocity.

In the girls, we found a significant positive correlation between handwriting quality and reaction time (r = 0.48, *p* < 0.05), vertical size (r = 0.43, *p* < 0.05), horizontal size (r = 0.39, p = *p* < 0.05), road length (r = 0.46, *p* < 0.05), and average absolute velocity (r = 0.41, *p* < 0.05). Thus, a high handwriting quality score was associated with a longer reaction time, a larger vertical size, a larger horizontal size, road length, and a greater average absolute velocity.

For handwriting speed, we found no correlations with any of the process features, except for a significant, positive correlation with average absolute velocity (r = 0.46, *p* < 0.05). Thus, a high handwriting speed was associated with a high average absolute velocity.

The present study aimed to analyze the relationship between the quality and speed of handwriting and the process characteristics of the handwriting of children in the second grade of elementary school with regard to age and gender.

In general terms, a poorer handwriting quality was associated with a higher number of strokes, reaction time, duration, vertical size, horizontal size, road length, and average absolute velocity in these groups. Conversely, a better handwriting quality was associated with a lower relative pen-down duration and average pen pressure in these groups. In addition, a higher handwriting speed was associated with a higher average absolute velocity in these groups. 

## 4. Discussion

### 4.1. Differences between Age Groups in Handwriting Quality and Speed and in Process Characteristics of Handwriting

Regarding the results, no significant differences were found between the age groups in handwriting quality and speed. According to previous studies, handwriting quality and speed can be expected to increase with age [7,12,14,15,16], but we did not observe that in the present study. Handwriting development depends on interactions between individual factors, the task, and the environment, and it is strongly influenced by exposure to instruction and practice [16,17]. Therefore, the fact that the children of both age groups were in the second grade and had been exposed to instruction and handwriting practice at the same time may have blurred possible differences in handwriting quality and handwriting speed with regard to age.

There were also no significant differences between the age groups in the process characteristics of handwriting. Despite the lack of consensus, previous studies in this area report the absence of any relationship between road length and age [9] and between handwriting pressure and age [9,38], which is in line with the findings of the present study. Considering the results of previous studies [6,9,35,37,38,39,40,41,50], we would have expected significant relationships between the other process characteristics of handwriting and age. However, we could not verify this. The large difference in the number of children in both age groups may explain the absence of age differences.

### 4.2. Differences between Gender Groups in Handwriting Quality and Speed and the Process Characteristics of Handwriting

With regard to handwriting quality and speed, we did not find significant differences between both genders. This is in line with previous studies [19,26,27].

Regarding gender differences in the process characteristics of handwriting, the boys showed a higher number of strokes and a lower slant than the girls, suggesting a movement with less fluency (automaticity) during handwriting. This finding seems inconsistent with that of Li et al. [9], who found that girls show a lower (i.e., less upright) handwriting slant than boys. This could be attributed to cultural influence, since [9] involved Chinese children, while, in Western societies, girls spend more time on fine motor tasks than boys. A greater slant angle (i.e., more upright handwriting) requires greater finger control and left-to-right wrist progression, while a lower slant angle (i.e., more rightward-slanted handwriting) requires a greater control of wrist movements and left-to-right arm movements [9].

Regarding other handwriting process characteristics, no gender differences were found, which is in line with other studies by Kushi et al. [42].

### 4.3. Correlations between the Handwriting Product and the Process Characteristics of Handwriting

The present study’s results show no significant correlations between handwriting quality and handwriting speed in the total population of participants and per age or gender group. Although there is no consensus, similar results have been found in several previous studies [18,19,20,21]. In the study by Weintraub and Graham [23], contradictory results were found, possibly due to the time constraints imposed during handwriting, while in the study by Volman et al. [10], the finding of an absence of associations may have arisen because the sample size in that study was smaller than that in this study.

We observed significant correlations between handwriting quality and the number of strokes, reaction time, duration, vertical size, horizontal size, path length, relative pen-down duration, average pen pressure, and average absolute velocity in the total population and in the 7-year-old group. Significant correlations were also found between handwriting quality and the vertical size and the average absolute velocity in the boys’ group and between handwriting quality and the reaction time, vertical size, horizontal size, road length, and average absolute velocity in the girls’ group.

Our result that a poorer handwriting quality is associated with larger vertical or horizontal sizes, i.e., larger handwriting, agrees with that of Vaivre-Douret et al. [37]. The size of the letters is thus an important indicator of handwriting quality [15,23,32].

Our finding that children with a poorer handwriting quality show longer reaction times may be the consequence of needing more time for the motor planning of handwriting, for example, to sequence the movements to be performed, to select the muscles to be activated, and to program the forces and durations to be exerted by the muscles. According to Thon [51], reaction time during handwriting is an important indicator of the complexity of the cognitive processes underlying response selection and motor programming. Longer reaction times are associated with more complex motor programs. Therefore, the more complex the words that are to be written, or the less familiar the words are as part of the children’s lexicon, the longer the time that elapses between perceiving and reading the words to be written and writing them by hand [47].

We found that worse handwriting quality is associated with a higher average absolute pen velocity. This was to be expected if we consider that pen velocity is the ratio of stroke size over stroke duration. We had already reported that an increased stroke size is strongly associated with worse handwriting quality, while an increased stroke duration has a slightly weaker association. According to Vaivre-Douret et al. [37], a worse handwriting quality was associated with a longer stroke duration, which can be explained by the fact that children need more time for handwriting motor planning, since their movements are less automatic and fluid and cost them more time. According to Albaret et al. [28], handwriting movements are initially slow and very dependent on sensory feedback, and then they become progressively faster and more fluid thanks to greater automaticity, i.e., more mature and proactive motor control [52]. This seems counterintuitive because, according to the Fitts’ law paradigm, there is a trade-off between movement duration and precision [53].

The BHK test defines handwriting speed as the number of letters written within 5 min. Our results failed to show a significant correlation between handwriting quality and handwriting speed, although Gosse et al. [22] and Weintraub and Graham [23] did report such a correlation. According to Gosse et al. [22], the speed with which one handwrites decreases when the objective is to write as neatly and legibly as possible. Conversely, accelerating the speed at which one produces letters will have a negative effect on readability [22].

However, in terms of the number of strokes, the literature argues that nonproficient handwriters produce a significantly larger number of strokes than proficient handwriters [54,55]: a poorer handwriting quality is associated with a greater number of strokes. This suggests worse in motor automation in these cases [56], which agrees with the results found in our study.

As expected, a significant correlation was also found between handwriting quality and relative pen-down duration. A worse handwriting quality was associated with a lower relative pen-down duration, which is in line with the study by Gargot et al. [8].

Another interesting finding was that a poorer handwriting quality was associated with less handwriting pressure. This result disagrees with that of some studies, which argue that, traditionally, handwriting pressure reflects the tension in the elbow, arm, and hand muscles during handwriting and that this muscle tension decreases as children become more proficient in handwriting [57]. According to previous studies, handwriting pressure is an important predictor of handwriting difficulties (dysgraphia), implying that a greater handwriting pressure is associated with a poorer handwriting quality and speed [8,36,55,58,59,60]. Evidence also suggests that the more mature (fluid and automatic) the handwriting gesture, the more easily the child can control it, for example, in terms of regulating the handwriting pressure applied [37]. In this study, the children with a poorer handwriting quality may have been afraid to apply pressure while writing on a digitizing tablet, as it is a different handwriting surface than usual (paper), which may have reduced the handwriting pressure.

Like Vaivre-Douret et al. [37], we found a significant correlation between the handwriting speed measured using the BHK and the average absolute velocity, as children who had a higher average absolute velocity wrote a larger number of characters from the BHK text.

Finally, no significant correlations were found between quality and the speed of handwriting, or between quality and the slant of handwriting, which is in line with the study of Gargot et al. [8].

## 5. Limitations and Future Directions

The current study needs to be interpreted with consideration of both its strengths and limitations. Firstly, the sample size of the 8-year-old group is much smaller than that of the 7-year-old group, because there were fewer 8-year-olds in the second grade of elementary school. These children benefited from delayed school entry due to age. Second, the sample size may have been insufficient to detect differences between age and gender groups and/or to detect significant associations in some variables. Many parents (73) did not consent to the participation of their children in this study, which may have introduced an unwanted selection of participants. In addition, three children were excluded from the present study due to unexpected failures in the MovAlyzeR^®^ system, which further limited the sample size. Finally, the small number of studies with the same objectives and assessment instruments made it difficult to compare the results.

Although our methodology is still expensive and time-consuming, it is considered a starting point for testing a larger, more representative population of participants in the future in order to expand and strengthen our preliminary findings. In addition, similar studies focusing on other age groups and comparing children with and without dysgraphia and the inclusion of other variables related to the characteristics of the handwriting process (e.g., electroencephalography) will be encouraged. Moreover, including an analysis of the context in which students learn to write [61] will allow us to extract new combinations of features using an artificial neural network (ANN), which will help to improve the early classification of children who are at risk of developing dysgraphia versus children who are not at risk [46].

## 6. Conclusions and Implications for Practice

Our findings provide evidence that the process characteristics of handwriting are similar in the age groups and genders studied, except for the number of strokes (which is higher in boys) and the slant (which is higher in girls). Furthermore, our findings provide evidence that handwriting quality and speed significantly correlate with various process characteristics of handwriting (e.g., the number of strokes, reaction time, duration, relative pen-down duration, average pen pressure, vertical and horizontal sizes, road length, and average absolute velocity) across the ages and genders studied. The results suggest that digitizing tablets are a valuable tool to identify handwriting process difficulties and that handwriting intervention programs should exploit the opportunity to work on these skills. To the best of our knowledge, there is no research taking the approach of both product and process assessment using the BHK, although the BHK is an instrument frequently used in clinical assessments. We realize that more research will be necessary to confirm and expand on our findings.

This research provides a promising step towards quantifying the development of handwriting quality and the speed and process characteristics of the handwriting of children as a function of age and gender. This research could lead to methods for the early identification of neuromotor difficulties that can lead to dysgraphia and, thus, eventually to the remediation or prevention of handwriting difficulties. In addition, the findings of the present study are important from a developmental perspective, as they provide promising contributions to the construction of normative values in the process characteristics of the handwriting of elementary school children. This contribution will be fundamental to achieving precision and efficiency in diagnoses by therapists. Furthermore, examining the relationships between the variables studied will help teachers, educators, and therapists to develop more effective interventions.

## Figures and Tables

**Table 1:** Handwriting product and process characteristics according to age and gender.

	Age 7 *n* = 43	Age 8 *n* = 14	*p*-Value	Boys *n* = 22	Girls *n* = 35	*p*-Value
HQ ^a^	21.72 ± 5.84	22.36 ± 6.13	0.73	22.95 ± 6.85	21.20 ± 5.14	0.28
HS ^a^	145 ± 35	135 ± 51	0.40	141 ± 30	144 ± 44	0.83
NS ^b^	18.0 (16)	22.5 (9.00)	0.38	23.0 (13)	17.0 (17)	0.04 *
RT ^b^	2.39 (3.52)	2.94 (3.86)	0.88	2.84 (3.64)	2.32 (3.55)	0.17
DT ^b^	0.27 (0.11)	0.26 (0.18)	0.58	0.28 (0.12)	0.27 (0.11)	0.60
RPDD ^b^	0.12 (0.38)	0.13 (1.00)	0.60	0.13 (0.53)	0.13 (0.38)	0.97
APP ^a^	401 ± 145	363 ± 202	0.45	361 ± 164	410 ± 157	0.27
VS ^b^	0.17 (0.13)	0.23 (0.22)	0.73	0.17 (0.14)	0.19 (0.16)	1.00
HZS ^b^	0.12 (0.08)	0.14 (0.13)	0.52	0.12 (0.10)	0.12 (0.08)	0.86
RL ^b^	0.28 (0.18)	0.39 (0.43)	0.40	0.29 (0.31)	0.30 (0.31)	0.76
SL ^b^	1.36 (0.22)	1.30 (0.27)	0.53	1.27 (0.23)	1.36 (0.22)	0.03 *
AAV ^a^	1.06 ± 0.51	1.03 ± 0.58	0.86	1.02 ± 0.56	1.08 ± 0.51	0.70

Note: * Significant at *p* < 0.05. ^a^ Values expressed in mean ± standard deviation; *p*-values of independent-samples *t*-test. ^b^ Values expressed in median (interquartile range); *p*-values of Mann–Whitney U Test. HQ = handwriting quality; HS = handwriting speed; NS = number of strokes; RT = reaction time; DT = duration; RPDD = relative pen-down duration; APP = average pen pressure; VS = vertical size; HZS = horizontal size; RL = road length; SL = slant; AAV = average absolute velocity.

**Table 2:** Correlations between handwriting production and process characteristics for the 7-year-old and 8-year-old groups and the total population.

	HQ						HS					
	Age 7 n = 43		Age 8 n = 14		Total Population n = 57		Age 7 n = 43		Age 8 n = 14		Total Population n = 57	
	*r*	*p*	*r*	*p*	*r*	*p*	*r*	*p*	*r*	*p*	*r*	*p*
HQ	1		1		1		−0.14	0.38	−0.10	0.75	−0.19	0.17
HS	−0.14	0.38	−0.10	0.75	−0.19	0.17	1		1		1	
NS	0.35	0.04 *	−0.21	0.59	0.26	0.10	−0.06	0.75	−0.03	0.95	−0.11	0.51
RT	0.53	0.001 **	0.01	0.98	0.38	0.02 *	−0.20	0.26	−0.22	0.58	−0.23	0.15
DT	0.45	0.01 *	0.06	0.88	0.32	0.04 *	−0.16	0.38	0.27	0.49	−0.05	0.75
RPDD	−0.40	0.02 *	0.00	1.00	−0.30	0.06	0.10	0.60	0.00	1.00	0.10	0.54
APP	−0.38	0.03 *	0.27	0.48	−0.25	0.12	−0.04	0.83	0.25	0.52	0.00	0.99
VS	0.63	0.00001 **	0.15	0.70	0.50	0.0001 **	0.09	0.64	0.17	0.67	0.08	0.62
HZS	0.61	0.0001 **	−0.03	0.95	0.43	0.01 **	0.07	0.71	0.28	0.46	0.16	0.33
RL	0.64	0.00001 **	0.11	0.78	0.50	0.0001 **	−0.02	0.92	0.15	0.70	0.06	0.71
SL	−0.02	0.91	−0.34	0.38	−0.07	0.69	0.12	0.53	0.30	0.43	0.17	0.29
AAV	0.56	0.0001 **	0.07	0.86	0.49	0.001 **	0.21	0.25	0.42	0.27	0.24	0.14

Note: * Significantly positive at *p* < 0.05 or significantly negative at *p* < 0.05. ** Significant at *p* < 0.01. *p*-values of Spearman’s correlation. HQ = handwriting quality; HS = handwriting speed; NS = number of strokes; RT = reaction time; DT = duration; RPDD = relative pen-down duration; APP = average pen pressure; VS = vertical size; HZS = horizontal size; RL = road length; SL = slant; AAV = average absolute velocity.

**Table 3:** Correlations between the handwriting product and the process characteristics for each gender group.

	HQ				HS			
	Boys n = 22		Girls n = 35		Boys n = 22		Girls n = 35	
	r	*p*	r	*p*	r	*p*	r	*p*
HQ	1		1		−0.31	0.16	−0.12	0.49
HS	−0.31	0.16	−0.12	0.49	1		1	
NS	0.10	0.73	0.31	0.13	−0.01	0.98	−0.10	0.62
RT	0.17	0.55	0.48	0.01 *	−0.18	0.52	−0.24	0.24
DT	0.38	0.16	0.24	0.25	−0.10	0.73	0.04	0.85
RPDD	−0.38	0.17	−0.18	0.38	0.18	0.52	0.08	0.70
APP	−0.25	0.37	−0.17	0.42	0.04	0.88	−0.08	0.71
VS	0.52	0.04 *	0.43	0.03 *	−0.15	0.59	0.27	0.19
HZS	0.45	0.09	0.39	0.05 *	−0.30	0.29	0.39	0.05
RL	0.42	0.12	0.46	0.02 *	−0.14	0.63	0.25	0.23
SL	0.00	1.00	−0.06	0.78	−0.25	0.37	0.27	0.19
AAV	0.57	0.03 *	0.41	0.04 *	−0.21	0.45	0.46	0.02 *

Note: * Significant at *p* < 0.05. *p*-values of Spearman’s correlation. HQ = handwriting quality; HS = handwriting speed; NS = number of strokes; RT = reaction time; DT = duration; RPDD = relative pen-down duration; APP = average pen pressure; VS = vertical size; HZS = horizontal size; RL = road length; SL = slant; AAV = average absolute velocity.

**Figure A1:**
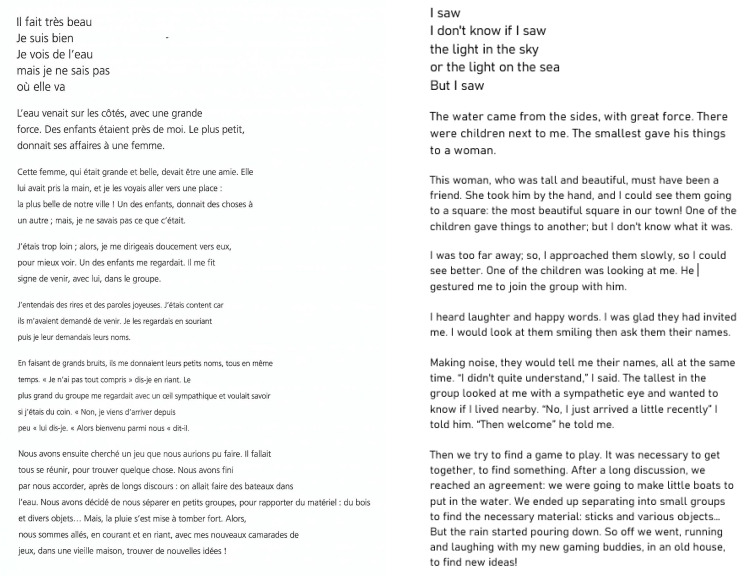
The French BHK text [15] (left panel) used to generate the cross-culturally adapted Portuguese version (shown in Figure 2) and the literal translation of the Portuguese version into English (right panel).

## Data Availability

The data presented in this study are available on request from the corresponding author.

## References

[1] Biotteau M., Danna J., Baudou E., Puyjarinet F., Velay J.-L., Albaret J.-M., Chaix Y. (2019). Developmental coordination disorder and dysgraphia: Signs and symptoms, diagnosis, and rehabilitation. Neuropsychiatr. Dis. Treat..

[2] McCloskey M., Rapp B. (2017). Developmental dysgraphia: An overview and framework for research. Cogn. Neuropsychol..

[3] McMaster E., Roberts T. (2016). Handwriting in 2015: A main occupation for primary school–aged children in the classroom?. J. Occup. Ther. Sch. Early Interv..

[4] Alhusaini A.A., Melam G.R., Buragadda S. (2016). Short-term sensorimotor-based intervention for handwriting performance in elementary school children. Pediatr. Int..

[5] Lachaux-Parker C. (2012). Troubles de l’écriture et psychomotricité. Rev. Francoph. d’Orthoptie.

[6] Accardo A.P., Genna M., Borean M. (2013). Development, maturation and learning influence on handwriting kinematics. Hum. Mov. Sci..

[7] Feder K.P., Majnemer A. (2007). Handwriting development, competency, and intervention. Dev. Med. Child Neurol..

[8] Gargot T., Asselborn T., Pellerin H., Zammouri I., Anzalone S.M., Casteran L., Johal W., Dillenbourg P., Cohen D., Jolly C. (2020). Acquisition of handwriting in children with and without dysgraphia: A computational approach. PLoS ONE.

[9] Lin Q., Luo J., Wu Z., Shen F., Sun Z. (2015). Characterization of fine motor development: Dynamic analysis of children’s drawing movements. Hum. Mov. Sci..

[10] Volman M.J.M., van Schendel B.M., Jongmans M.J. (2006). Handwriting Difficulties in Primary School Children: A Search for Underlying Mechanisms. Am. J. Occup. Ther..

[11] Schneck C., Amundson S., Case-Smith J., O’Brien J. (2010). Prewriting and Handwriting Skills. Occupational Therapy for Children.

[12] Van Hoorn J.F., Maathuis C.G.B., Hadders-Algra M. (2013). Neural correlates of paediatric dysgraphia. Dev. Med. Child Neurol..

[13] Klein S., Guiltner V., Sollereder P., Cui Y. (2011). Relationships Between Fine-Motor, Visual-Motor, and Visual Perception Scores and Handwriting Legibility and Speed. Phys. Occup. Ther. Pediatr..

[14] Overvelde A., Hulstijn W. (2011). Handwriting development in grade 2 and grade 3 primary school children with normal, at risk, or dysgraphic characteristics. Res. Dev. Disabil..

[15] Charles M., Soppelsa R., Albaret J.-M. (2008). BHK: Échelle d’Evaluation Rapide de l’écriture Chez l’Enfant.

[16] Ziviani J., Wallen M., Henderson A., Pehoski C. (2006). The Development of Graphomotor Skills. Hand Function in the Child: Foundations for Remediation.

[17] Kaiser M.-L., Soppelsa R., Albaret J.-M., Albaret J.-M., Kaiser M.-L., Soppelsa R. (2013). Aspects développementaux. Troubles de l’Ecriture chez l’Enfant.

[18] Graham S., Berninger V., Weintraub N., Schafer W. (1998). Development of Handwriting Speed and Legibility in Grades 1–9. J. Educ. Res..

[19] Karlsdottir R., Stefansson T. (2002). Problems in Developing Functional Handwriting. Percept. Mot. Ski..

[20] Schwellnus H., Carnahan H., Kushki A., Polatajko H., Missiuna C., Chau T. (2012). Effect of Pencil Grasp on the Speed and Legibility of Handwriting in Children. Am. J. Occup. Ther..

[21] Wicki W., Lichtsteiner S.H., Geiger A.S., Müller M. (2014). Handwriting Fluency in Children. Swiss, J. Psychol..

[22] Gosse C., Parmentier M., Van Reybroeck M. (2021). How Do Spelling, Handwriting Speed, and Handwriting Quality Develop During Primary School? Cross-Classified Growth Curve Analysis of Children’s Writing Development. Front. Psychol..

[23] Weintraub N., Graham S. (1998). Writing legibly and quickly: A study of children’s ability to adjust their handwriting to meet the common classroom demands. Learn. Disabil. Res. Pract..

[24] Vlachos F., Bonoti F. (2006). Explaining age and sex differences in children’s handwriting: A neurobiological approach. Eur. J. Dev. Psychol..

[25] Yu T.-Y., Howe T.-H., Hinojosa J. (2012). Contributions of Haptic and Kinesthetic Perceptions on Handwriting Speed and Legibility for First and Second Grade Children. J. Occup. Ther. Sch. Early Interv..

[26] Kaiser M.-L. (2012). Qualité et vitesse de l’écriture chez l’enfant. L’Educateur.

[27] Kaiser M.-L., Albaret J.-M., Doudin P.-A. (2009). Relationship Between Visual-Motor Integration, Eye-Hand Coordination, and Quality of Handwriting. J. Occup. Ther. Sch. Early Interv..

[28] Albaret J.-M., Danna J., Soppelsa R., Kaiser M.-L., Albaret J.-M., Kaiser M.-L., Soppelsa R. (2013). Définitions et modèles. Troubles de l’Ecriture chez l’Enfant: Des Modèles à l’Intervention.

[29] Danna J., Velay J., Albaret J.-M., Pinto S., Sato M. (2016). Dysgraphies. Traité de Neurolinguistique: Du Cerveau au Langage.

[30] Rosenblum S., Weiss P.L., Parush S. (2003). Product and Process Evaluation of Handwriting Difficulties. Educ. Psychol. Rev..

[31] Soppelsa R., Abizeid C., Chéron A., Laurent A., Danna J., Albaret J.-M., Albaret J.-M., Abizeid C.-M., Soppelsa R. (2016). Dysgraphies et Rééducation Psychomotrice: Données Actuelles. Les Entretiens de Bichat 2016.

[32] Rosenblum S., Weiss P.L., Parush S. (2004). Handwriting evaluation for developmental dysgraphia: Process versus product. Read. Writ..

[33] Rosenblum S., Livneh-Zirinski M. (2008). Handwriting process and product characteristics of children diagnosed with developmental coordination disorder. Hum. Mov. Sci..

[34] Hamstra-Bletz E., de Bie J., den Brinker B.P.L.M. (1998). Beknopte Beoordelingsmethode voor Kinderhandschriften.

[35] Accardo A., Genna M., Borean M., Saule B. Parametric analysis of handwriting in school-age children. Proceedings of the 7th European Symposium on Biomedical Engineering (ESBME).

[36] Rückriegel S., Burghardt R., Ehrlich S., Blankenburg F., Driever P.H. (2008). Development of kinematic properties of drawing and handwriting movements in healthy children and adolescents. Neuropediatrics.

[37] Vaivre-Douret L., Lopez C., Dutruel A., Vaivre S. (2021). Phenotyping features in the genesis of pre-scriptural gestures in children to assess handwriting developmental levels. Sci. Rep..

[38] Silveri G., De Dea F., Perrone I., Accardo A. (2019). Influence of Dysgraphia on Kinematic Characteristics of Handwriting in Italian Primary School Children. IFMBE Proc..

[39] Barrientos P. (2016). Handwriting Development in Spanish Children With and Without Learning Disabilities: A Graphonomic Approach. J. Learn. Disabil..

[40] Jia L., Zhang C., Gao L., Sun Z., Zheng W., Qi H. Kinematic Analysis of Drawing Movements in Chinese Primary Schoolchildren. Proceedings of the CNIOT2020: 2020 International Conference on Computing, Networks and Internet of Things.

[41] Zengwu S., Qiushi L., Jianfei L., Tingting R., Zhongcheng W. (2014). Characterization of drawing movement as schooling advances in primary school. Comput. Model. New Technol..

[42] Kushki A., Schwellnus H., Ilyas F., Chau T. (2011). Changes in kinetics and kinematics of handwriting during a prolonged writing task in children with and without dysgraphia. Res. Dev. Disabil..

[43] Genna M., Accardo A. (2011). Gender and Age Influence in Handwriting Performance in Children and Adolescents. IFMBE.

[44] Askvik E.O., Van Der Weel F.R., Van Der Meer A.L.H. (2020). The Importance of Cursive Handwriting Over Typewriting for Learning in the Classroom: A High-Density EEG Study of 12-Year-Old Children and Young Adults. Front. Psychol..

[45] Feng L., Lindner A., Ji X., Joshi R.M. (2019). The roles of handwriting and keyboarding in writing: A meta-analytic review. Read. Writ..

[46] Kulik S.D. (2015). Neural Network Model of Artificial Intelligence for Handwriting Recognition. J. Theor. Appl. Inf. Technol..

[47] Matias A. (2016). Study of Graphomotor Skills in Children in the 3rd Year of Schooling, in the Lisbon Region. Ph.D. Thesis.

[48] (2020). Neuroscript, Glossary. https://neuroscript.net/help/lexicon.html.

[49] Marôco J. (2018). Análise Estatística com o SPSS Statistics.

[50] Gerth S., Klassert A., Dolk T., Fliesser M., Fischer M.H., Nottbusch G., Festman J. (2016). Is Handwriting Performance Affected by the Writing Surface? Comparing Preschoolers’, Second Graders’, and Adults’ Writing Performance on a Tablet vs. Paper. Front. Psychol..

[51] Thon B., Albaret J.-M., Soppelsa R. (2019). Approche comportementale et cognitive de la motricité humaine : Concepts, méthodes et modèles. Précis de Rééducation de la Motricité Manuelle.

[52] Smits-Engelsman B., Wilson P., Westenberg Y., Duysens J. (2003). Fine motor deficiencies in children with developmental coordination disorder and learning disabilities: An underlying open-loop control deficit. Hum. Mov. Sci..

[53] Fitts P.M. (1954). The information capacity of the human motor system in controlling the amplitude of movement. J. Exp. Psychol..

[54] Falk T.H., Tam C., Schwellnus H., Chau T. (2010). Grip Force Variability and Its Effects on Children’s Handwriting Legibility, Form, and Strokes. J. Biomech. Eng..

[55] Rosenblum S., Dror G. (2016). Identifying Developmental Dysgraphia Characteristics Utilizing Handwriting Classification Methods. IEEE Trans. Hum. Mach. Syst..

[56] Accardo A., Costa F., Perrone I. (2017). The Influence of the Spatio-Temporal Terzi Treatment on the Kinematics of Cursive Writing of Dysgraphic Subjects. IEEE Trans. Hum. Mach. Syst..

[57] Di Brina C., Niels R., Overvelde A., Levi G., Hulstijn W. (2008). Dynamic time warping: A new method in the study of poor handwriting. Hum. Mov. Sci..

[58] Asselborn T., Chapatte M., Dillenbourg P. (2020). Extending the Spectrum of Dysgraphia: A Data Driven Strategy to Estimate Handwriting Quality. Sci. Rep..

[59] Mekyska J., Faundez-Zanuy M., Mzourek Z., Galaz Z., Smekal Z., Rosenblum S. (2017). Identification and Rating of Developmental Dysgraphia by Handwriting Analysis. IEEE Trans. Hum. Mach. Syst..

[60] Tseng M.H., Cermak S.A. (1993). The Influence of Ergonomic Factors and Perceptual–Motor Abilities on Handwriting Performance. Am. J. Occup. Ther..

[61] Graham S. (2018). A Revised Writer(s)-Within-Community Model of Writing. Educ. Psychol..

